# α-Carotene and β-Carotene Content in Raw and Cooked Pulp of Three Mature Stage Winter Squash “Type Butternut”

**DOI:** 10.3390/foods4030477

**Published:** 2015-09-18

**Authors:** Fernanda Zaccari, Giovanni Galietta

**Affiliations:** 1 Postharvest Fruits and Vegetables, Faculty of Agronomy, Universidad de la República, Av. Eugenio Garzón 780, CP12900 Montevideo, Uruguay; 2 Food Technology, Faculty of Agronomy, Universidad de la República, Av. Eugenio Garzón 780, CP12900 Montevideo, Uruguay; E-Mail: galietta.giovanni@gmail.com

**Keywords:** *Cucurbita moschata*, ripening, cooked pulp, color, carotenes

## Abstract

Winter squash “type butternut” is harvested in physiological ripening for better commercial distribution, when sensory and/or nutritional quality is not optimum for consumption. The objective of this study was to quantify the content of α-carotene, β-carotene, color and dry matter in the pulp of raw and microwave-cooked winter squash “type butternut” (variety CosmoF1) in three states of commercial maturity. Immature, mature, and very mature fruit, defined at the time of the harvest by the percentage of orange peel and green stalk, were evaluated. The highest concentration of carotenes (α-carotene + β-carotene) in mg.100 g^−1^ pulp wet basis was found in very mature fruits (31.96 mg), followed by mature fruits (24.65 mg), and immature fruits (18.82 mg). Microwave cooking caused the loss of β-carotene (28.6% wet basis) and α-carotene (34.1%). Cooking promote a greater reduction of α-carotene in immature (40.3%) and mature (34.5%) fruits. The ratio of β-carotene and α-carotene content increased with commercial maturity from 0.93 for immature fruits to 1.0 for very mature fruit, with higher ratio in cooked pulp (1.04) *vs.* raw pulp (0.96).

## 1. Introduction

Winter squash “butternut types” (*Cucurbita moschata* Duch.) are fruits harvested in stages during the summer season and preserved for 3–6 months [1]. “Butternut squash” varieties consumed in Uruguay have orange colored peel and flesh. The change in peel color from green to orange is used as an indicator of fruit maturity and harvesting time. The yellow and orange color, on peel and pulp, are attributed to carotenoids contents [2,3,4]. Carotenoids are natural pigments, some of the precursors of vitamin A, such as α and β-carotene, have antioxidant properties and protect against the negative effects of light [3,4,5]. The chemistry and biology of the carotenoids is well known, and these compounds are studied for their positive nutrition and human health properties. Carotenoids are associated with different physiological processes including vision, the absorption of iron, homeostasis, immune system, dyslipidemia, inflammation, insulin resistance, and risk reduction for various types of cancer [3,4,5,6]. “Butternut type” squash is harvested during physiological maturity, very close to the optimum organoleptic quality [2,7,8]. The organoleptic state of maturity is associated with the color, texture and flavor [8] also related to nutritional properties [5,6,7,8,9]. One of the main logistical problems for the marketing of squash is to harvest fruits of similar maturity stage, to consolidate homogeneous loads when long term shipping and/or prolonged storage is needed. The fruits are both marketed for fresh consumption as well as for industrial processing. Physical, chemical and nutritional characteristics can be modified by the fruit ripeness (in or outside the plant) the conditions and time of storage and the preparation form [9,10]. Loss of carotenoids during boiling or roasting of squash pulp depends on species, variety, maturity, and cooking procedure [9,10,11]. The objective of this study was to quantify the content of α-carotene and β-carotene in raw and cooked winter squash “type butternut” (*Cucurbita moschata* Duch.) variety CosmosF1 (Sakata), in three stages of maturity at the time of the commercial harvest.

## 2. Experimental Section

### 2.1. Plant Materials and Fruit Sample Preparation

**Figure 1:**
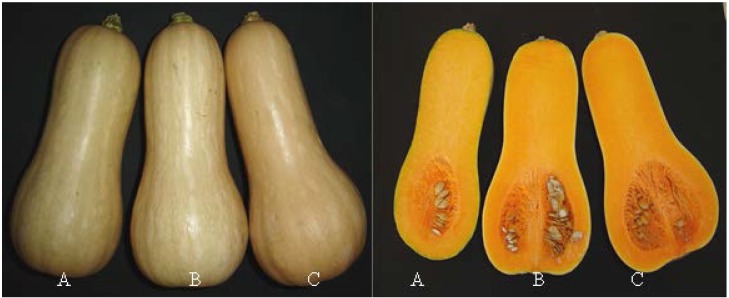
Stages of commercial maturity of the fruits. (A): immature; (B): mature; (C): very mature.

“Type butternut” squash variety CosmosF1 (Sakata) fruits were harvested in March (late Summer) from a crop located in latitude South 34°55′66″, Longitude West 55°90′98″. The commercial maturity of the winter squash was delimited at the time of the harvest according to the percentage of orange peel (N) and the green color of the peduncle (V), according to [12]. Three stages of maturity were defined: immature fruit (50%–60% N; 50% V), mature fruit (80%–90% N, 20% V), and very mature fruit (100% N, 0% V) (Figure 1).

The fruits were transferred to Post Harvest Fruits and Vegetables laboratory of the Faculty of Agronomy (Universidad de la República). Fruits without visible defects were washed with drinking water, disinfected with commercial sodium hypochlorite (100 mg·L^−1^, 5 min with agitation), dried with absorbent paper, and cut. Fruits were cut between the peduncle and the seminal cavity. The pulp around the seed cavity was discarded and two to three slices of pulp per fruit were extracted (10–12 cm in diameter and 3 cm thick), peeled, and cut into 3 cm^3^ per cubes. Cubes were divided into two groups: raw and cooked by microwave. Cooking was done by placing the cubes of flesh in warm water (45 °C; 1:1 water:pulp ratio) and inside a microwave oven (Kassel-KSMM20) set to 800 W and 4 min.

At the end point of cooking, temperature of the pulp was 52 to 56 °C as determined with a temperature probe (Digi-Sense, Vernon Hills, IL USA), and the cubes had a firm texture. The samples were kept in freezer (−20 °C) to perform the analysis.

### 2.2. β-Carotene and α-Carotene Content

In each stage of maturity, the carotenoids content was determined in raw and cooked pulp in triplicate. Carotenes extraction and quantification was carried out following the protocol and the HPLC equipment described by [13]. Standard β-carotene (≥95%) and carrot carotenoids (β:α 2:1, ≥95%) were purchased from Sigma-Aldrich Corp (St. Louis, MO, USA).

In each treatment the ratio between β-carotene and α-carotene content was calculated according to the following Formula (1):

(1) $$\frac{\text{β}}{\alpha }=\frac{\left[\text{β}-\text{carotene}\right]}{\left[\text{α}-\text{carotene}\right]}$$

where [α-carotene] and [β-carotene] are the concentrations of α and β-carotene measured by HPLC, respectively.

### 2.3. Color

The color of the raw and cooked pulp was determined in quadruplicate in each treatment and plot, using a colorimeter (Minolta CR10, Japan) to determine the CIELAB parameters (L*, a* and b*). The recommendations of the Commission Internationale de L’Eclariage [14] were followed: Standard illuminant D65 and 10° Standard Observer. Hue angle (*h_ab_*) and chroma (C*_ab_) were calculated using the following Formulas (2) and (3):

(2) hab=tan−1(b*a*)

(3) $$C_{ab}^{*}=\sqrt{{\left(a^{*}\right)}^{2}+{\left(b^{*}\right)}^{2}}$$

### 2.4. Percentage of Reduction of Carotenes by Cooking

For each stage of maturity, the percentage of reduction of carotenes by cooking was calculated using with the following Formula (4):

(4) $$\%\;ReductionCarotene=100-\frac{\left[Raw\;pulp\right]}{\left[Cooked\;pulp\right]}\times 100$$

where [*Raw pulp*] is the concentration of α and β-carotene in raw pulp measured by HPLC and [*Cooked pulp*] is concentration of αand β-carotene in cooked pulp.

### 2.5. Dry Matter Content

Dry matter content for each treatment was measured in duplicate by drying the samples in an oven with ventilation to constant weight at 105 °C. Data was expressed as percent dry matter (% MS), calculated with this Formula (5):

(5) $$\%MS=100\cdot \frac{M_{f}}{M_{i}}$$

where *M_f_* is the pulp mass after drying and *M_i_* is the initial pulp mass (before drying).

### 2.6. Experimental Design and Statistical Analysis

Experimental design consisted of completely randomized plots in a 3 × 2 factorial structure (factor maturity with three levels and factor preparation with two levels), with three plots per treatment (8 fruits; 10–15 kg per plot). The means were compared using the Tukey test (*p* ≤ 0.05) after analysis of variance showed significant treatment effects. Within each stage of maturity means for factor preparation (raw and cooked) were compared using the paired Student test (*p* ≤ 0.05%). The data were processed with the InfoStat (Version 2004, FCA, Córdoba, Argentine) statistical program.

## 3. Results and Discussion

### 3.1. Color and Content of β-Carotene and α-Carotene in Winter Squash

The content of β-carotene and α-carotene, in raw and cooked pulp increased with commercial fruit maturity. Statistical differences were found of both α-carotene and β-carotene, as well as the preparation form (Table 1). Cooking resulted in a reduction of β-carotene (*p* = 0.0016) and α-carotene (*p* = 0.0004). The content of β-carotene significantly dropped (33.4%) after cooking only for raw immature fruits. However, significant reductions in α-carotene content were observed after cooking immature (40.2%) and mature (34.5%) fruit pulps (Table 1). No significant interaction effect was found between the form of preparation (raw *vs.* cooked) and the ripeness of the fruit for both carotenes (Table 1).

**Table 1:** Total content of β-carotene and α-carotene (mg.100 g^−1^ wet basis) in raw and cooked pulp from winter squash at three stages of maturity.

	*β-Carotene*		*α-Carotene*	
	Raw	Cooked	Raw	Cooked
Immature	9.54 ± 0.61 bA	6.35 ± 0.62 bB	9.28 ± 0.31 bA	5.55 ± 0.54 bB
Mature	12.34 ± 0.47 abA	9.37 ± 0.91 abA	14.31 ± 0.67 abA	9.37 ± 0.65 bB
Very Mature	15.77 ± 1.61 aA	11.08 ± 0.83 aA	16.19 ± 1.79 aA	11.31 ± 0.89 aA
**Mains effects**	***p* Value (β)**		***p* Value (α)**	
Stage of Maturity (SM)	0.0016		0.0007	
Preparation (P)	0.0016		0.0004	
SM x P	0.6458		0.8421	

Mean ± SEM (*n* = 3). For β-carotene or α-carotene, lowercase letters in a column indicate differences between stages of maturity within the same form of preparation (Tukey ≤ 0.05). Uppercase letters in a row indicate differences between the ways of preparation for the same stage of mature (Student *t* ≤ 0.05).

Observed contents of α-carotene content of β-carotene in squash “type butternut”, variety CosmosF1, in all its stages of maturity were in agreement with results reported in other varieties of *Cucurbita moschata* “butternut type” [4,7,11,15]. A higher concentration of carotenes (α, β) were obtained in the very mature stage (31.96 mg·100 g^−1^ flesh wet basis) compared to immature raw fruits (18.32 mg·100 g^−1^ flesh wet basis), and with cooked pulp (22.29 mg·100 g^−1^ flesh wet basis) in very mature fruits and in immature fruits (11.90 mg·100 g^−1^ flesh wet basis). These results were consistent with visual observations of the color of the pulp. Fruits with greater stage of commercial maturity had a more intense orange color in the raw pulp (Figure 1). The hue (61.3°) and chrome (67.4) of the raw flesh of very ripe fruit (Table 2) were statistically different from the other maturity stages and demonstrated a higher concentration of pigments in fruits with advanced ripening. Cooking changed the color of the pulp at different stages of maturity of fruit, determining a lower chroma and hue value in raw pulp (Table 2). This effect was statistically significant in the pulp of immature and mature fruits. However, in the three stages of maturity studied, cooked pulp had lower lightness than raw pulp (Table 2).

**Table 2:** Lightness (L*), Chroma (C*_ab_) and Hue angle (*h_ab_*) color of raw and cooked pulp from squash at three stages of maturity.

	Immature	Mature	Very Mature
	Lightness (L*)		
Raw	69.4 ± 0.3 aA	68.5 ± 0.4 aA	65.8 ± 0.5 bA
Cooked	57.5 ± 1.5 aB	62.7 ± 1.6 aB	51.6 ± 1.5 bB
	***Hue Angle (h_ab_)***		
Raw	67.1 ± 0.6 aA	66.1 ± 0.3 aA	61.3 ± 0.6 bA
Cooked	73.2 ± 1.0 aB	67.8 ± 0.8 bA	65.2 ± 1.2 bB
	Chroma (C*_ab_)		
Raw	62.3 ± 0.3 bA	63.9 ± 1.4 bA	67.4 ± 0.8 aA
Cooked	50.7 ± 1.9 bB	59.8 ± 2.0 aA	49.5 ± 1.8 bB

Mean ± SEM (*n* = 12). For each color variable, same lowercase letters for the same row (Tukey *p* ≥0.05) and uppercase letters for the same column (*t*-Student *p* ≥ 0.05), indicate no statistical difference.

The factors determining the final content of carotenes in fruits and vegetables are not fully understood. There are studies how the expression of specific genes played a key role in the accumulation of carotenes and ultimately determined higher or lower carotene content during fruit development [4]. Zhang *et al.* [4] worked with *Cucurbita moschata*, Max variety, raw pulp, standard production practices; at harvest mature stage index described by weeks after pollination, report lutein but not alpha carotene. In our experimental conditions, *Cucurbita moschata*, Cosmos F1 variety, raw pulp, at harvest time, in three commercial mature stages described with color of the peel and stalk, lutein was not found. These results suggest, as others authors [4,7,9], that the profile and amount of carotenoids is determined by several factors such as genetic material as well as handling, environmental factors and process.

Early expression of genes encoding enzymes is a critical factor to regulate the accumulation of carotenoids [4,5,6,7,8,9,10,11,12,13,14,15,16]. However, [17] in watermelon indicated that there is no correlation between carotenoid biosynthesis gene expression and specific carotene accumulation. On the other hand, depending on the cooking conditions, carotenoids can be more or less affected, resulting in an increase or reduction of their concentration [18,19]. Furthermore, the highly unsaturated chemical structure of α and β-carotene, makes them susceptible to degradation by physical and chemical environmental factors, including oxygen, light, temperature, pH [5,6,7,8,9]. Oxidations of carotenoids as well as the formation of new compounds have been linked to changes in the instrumental color of food and its consequent effect on nutritional and/or sensorial attributes [8,19,20]. In our study of raw and cooked pulp of the CosmosF1 variety, isomers or other carotenoids detected in samples that probably help defining color were not analyzed.

A reduction in the concentration of carotenoids that has been observed could be associated with the loss of solutes in the cooking water, due time and temperature induced changes in plant tissue during cooking [10,11,12,13,14,15,16,17,18,19,20,21]. At the time of harvest, the variety CosmosF1 did not show significant difference in dry matter content after microwave cooking (*p* = 0.3911) or by the stage of commercial maturity of the fruits (*p* = 0.6874) (Table 3). Other metabolic and structural modifications, like starch or fiber, may determine the retention of carotenoids during cooking [5,6,7,8].

**Table 3:** Dry matter content (%) of raw and cooked winter squash pulp in three stages of maturity.

	Dry matter content (%)			*p* Value
	Immature	Mature	Very Mature	
Raw	8.75 ± 0.75	8.28 ± 0.29	8.60 ± 0.59	0.8403
Cooked	9.26 ± 0.53	8.83 ± 0.17	8.04 ± 0.14	0.1426
*p* Value	0.6779	0.2182	0.2131	
***Main Effects***	***p Value***			
Maturity stage (MS)	0.3911			
Preparation (P)	0.6874			
MS × P	0.6458			

Mean ± SEM (*n* = 6).

### 3.2. Ratio β:α

**Figure 2:**
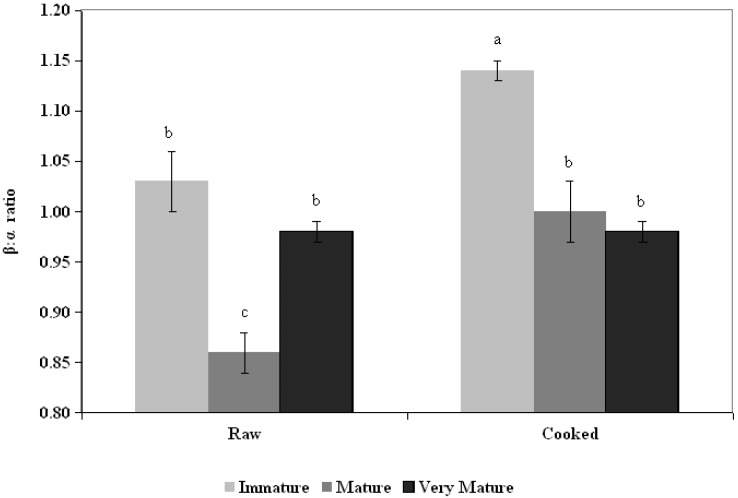
Relationship between β-carotene and α-carotene content, in every state of fruit maturity and preparation. Mean ± SEM (*n* = 3). Different letters in the column indicate that the treatments are statistically different. Main effects: state of maturity (SM) < 0.0001; Preparation (*p*) < 0.0001; SM × *p* = 0.0044.

The ratio of β-carotene to α-carotene content had values between 0.84 and 1.15 in the studied treatments, and significant interaction effect (*p* = 0.0044) was found between the two factors analyzed (maturity *vs.* preparation). Raw mature fruit exhibited a lower β:α ratio (0.86) *vs.* all other treatments and the cooked immature pulp (1.15), other similar treatments being different to each other (Figure 2).

As mentioned previously, the synthesis of carotenoids is a complex and continuous process where several genes express during the ripening and aging of the fruit are involved, and different variety and/or condition crop contribute for change de ratio of carotenes [4,5,6,7,8,9,10,11,12,13,14,15,16,17,18]. At the same time the form of cooking can differentially reduce the carotenes present in each stage of maturity of the fruit. Immature fruit probably has a composition in the metabolic and cellular structure different from the of more mature fruits [8], which can be determined during cooking that carotenoids are labile, removed or may have oxidations when exposed to aggressive environmental conditions [9,10,11,12,13,14,15,16,17,18,19,20,21].

### 3.3. Percentage Reduction of Carotenes

**Figure 3:**
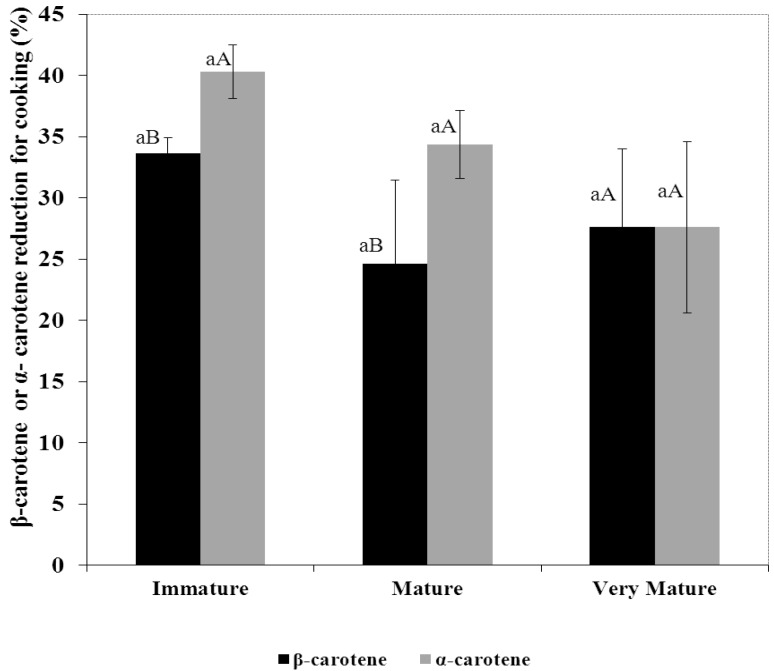
α-carotene and β-carotene reduction for cooking from winter squash pulp in three states of fruit maturity. Mean ± SEM (*n* = 3). For α-carotene or β-carotene, different lowercase letters indicates differences between states of maturity (Tukey, *p* ≤ 0.05). Different capital letters indicate differences between carotenoids to the same state of maturity (*t*-Student *p* ≤ 0.05).

The percentage reduction by the effect of cooking, both β-carotene (28.6%) and α-carotene (34.1%) had no statistical difference (*p* = 0.7701, *p* = 0.6507) between the states of commercial maturity. However, while the pulp of the fruits in the most advanced state of maturity (very mature) had a similar reduction of both carotenoids (27.6%), in mature and immature fruits, which lost more α-carotene than β-carotene (Figure 3).

Similar results in the differential retention of α-carotene and β-carotene were observed by [11] and [16] in squash (*Cucurbita moschata*), and [22] in carrot, where α-carotene presented lower retention rate than β-carotene. The index of the amount of retention of carotene in carrot samples was similar when they were prepared with similar time and temperature, and similar relation water/pulp cooking and cutting processes before cooking.

## 4. Conclusions

Commercial maturity determined the different content of carotenes at harvest time. Very mature fruit pulp showed higher concentration of carotenes (α, β) than mature and immature fruit pulps (31.96; 24.65 and 18.42 mg·100 g^−1^ pulp wet basis, respectively). Cooking using a microwave oven reduced the α-carotene (34.1%) and β-carotene (28.6%) content. Cooking loss was higher for α-carotene than β-carotene in immature and mature fruits. The greatest loss of α-carotene by cooking in a microwave oven occurred in the pulp of immature state fruits (40.3%). Cooking did not alter the dry matter content of any of the studied squash pulp. The ratio of β-carotene to α-carotene content increased during maturity doing the state of commercial maturity (0.93; 0.98; 1.0; immature, mature and very mature respectively) and was higher in the cooked pulp. The winter squash “type butternut” variety CosmoF1 is an important source of pro-vitamin A in both raw and cooked pulp and in the three states of commercial maturity evaluated.
